# Supplementary material for: Chromosome-scale genome assembly of a Japanese chili pepper landrace, Capsicum annuum ‘Takanotsume’
Source: DNA Res. 2022 Dec 25;30(1):dsac052. doi: 10.1093/dnares/dsac052 (PMC9886071; doi:10.1093/dnares/dsac052)
Supplement: dsac052_suppl_Supplementary_Data [file dsac052_suppl_supplementary_data.pdf]

Supplementary Information

Chromosome-scale genome assembly of a Japanese chili pepper landrace, *Capsicum annuum* 'Takanotsume'

Kenta Shirasawa[1*], Munetaka Hosokawa[2,3], Yasuo Yasui[4], Atsushi Toyoda[5], and Sachiko Isobe[1]

[1]Department of Frontier Research and Development, Kazusa DNA Research Institute, Kisarazu, Japan

[2]Department of Agriculture, Kindai University, Nara, Japan

[3]Agricultural Technology and Innovation Research Institute, Kindai University, Nara, Japan

[4]Graduate School of Agriculture, Kyoto University, Kyoto, Japan

[5]Advanced Genomics Center, National Institute of Genetics, Mishima, Japan

[*]To whom correspondence should be addressed:

Kenta Shirasawa

2-6-7 Kazusa-Kamatari, Kisarazu, Chiba 292-0818, Japan

Tel.: +81-438-52-3935

Fax: +81-438-52-3934

E-mail: shirasaw@kazusa.or.jp

Supplementary Table S1 Genome coverage of the assembled sequences of *Capsicum* lines.

Supplementary Table S2 BUSCO scores for the genome assemblies and predicted genes in *Capsicum* lines.

Supplementary Table S3 Gene IDs and annotations for the high confidence genes predicted in the 'Takanotsume' genome.

Supplementary Table S4 Annotation of variants detected among 13 *Capsicum* lines.

Supplementary Table S5 Genome positions of transposon insertion polymorphism across 13 *Capsicum* lines.

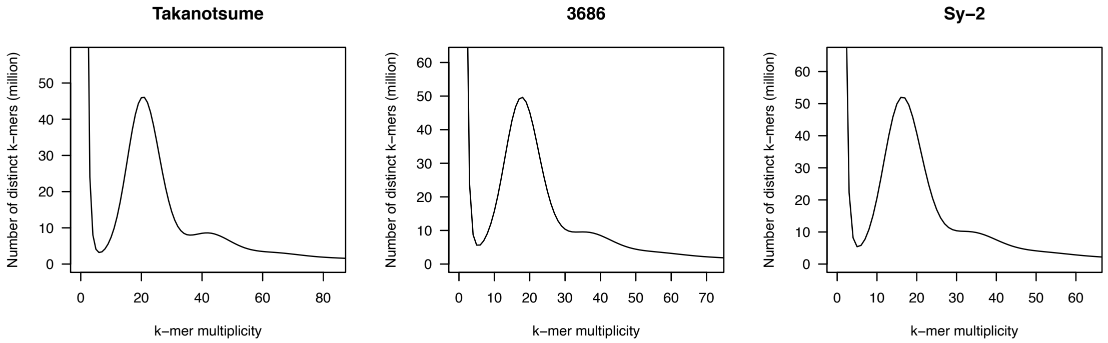

Supplementary Figure S1 Estimated genome sizes of *Capsicum annuum* landrace 'Takanotsume' and *C. chinense* lines '3686' and 'Sy-2', based on *k*-mer analysis ($k = 17$), with the given multiplicity values.

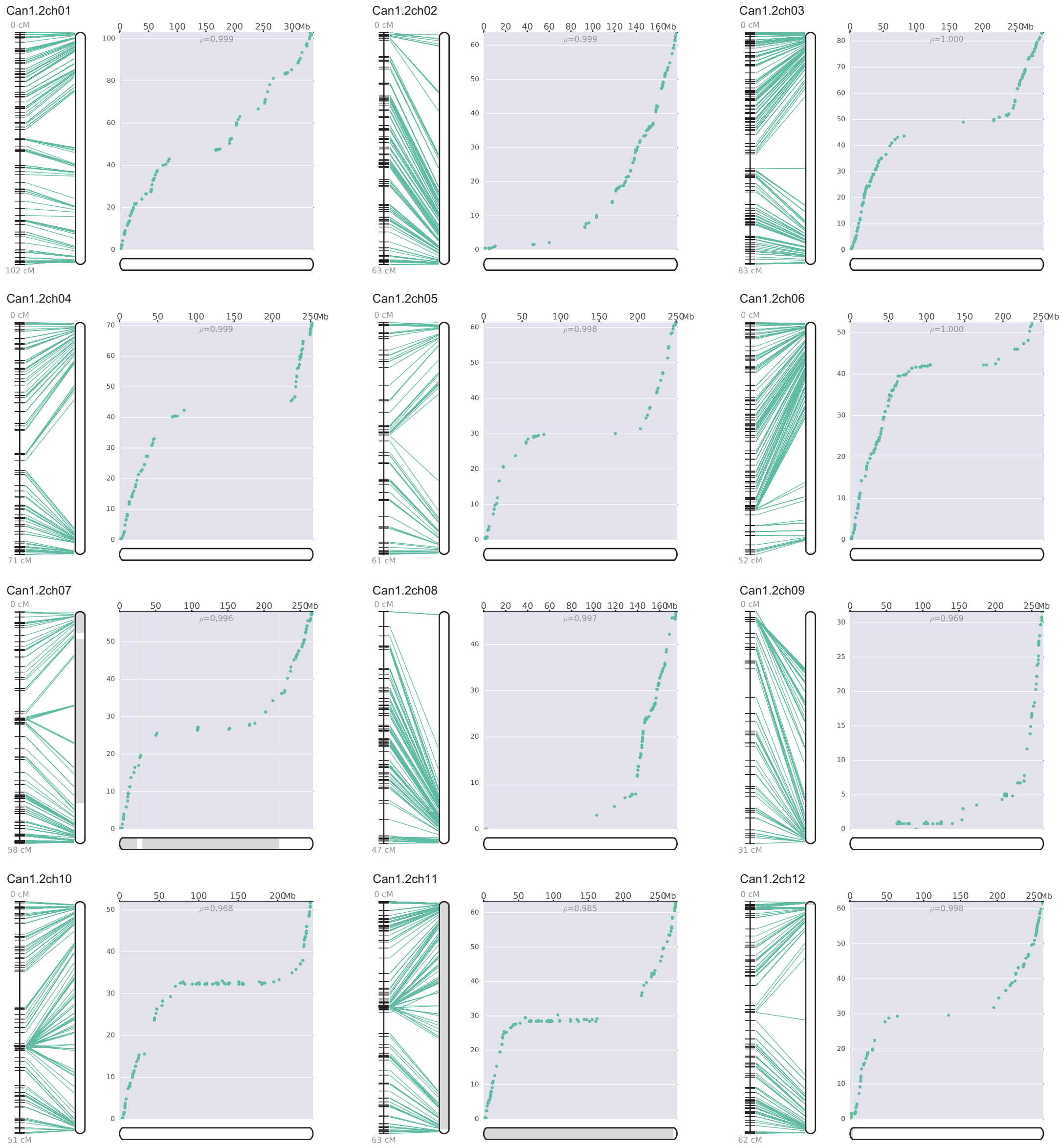

Supplementary Figure S2 Genetic and physical maps of the ‘Takanotsume’ genome.

Left: SNP loci on the genetic maps (vertical lines) and the physical maps (bars) are connected with horizontal lines. Right: Positions of the SNP loci are indicated by dots on the genetic maps (on the y axis, cM) and the physical maps (on the x axis, Mb).

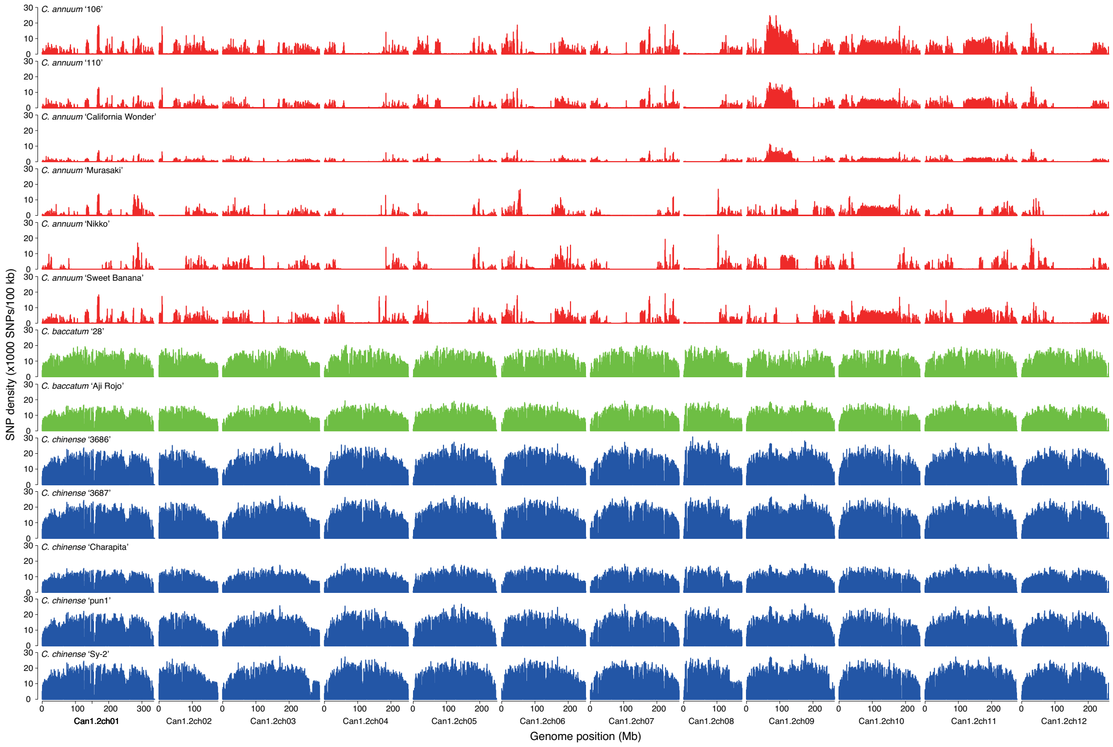

Supplementary Figure S3 SNP density across the 13 *Capsicum* lines, based on the comparison with the 'Takanotsume' genome.

C. annuum, *C. baccatum*, and *C. chinense* lines are shown in red, green, and blue, respectively.

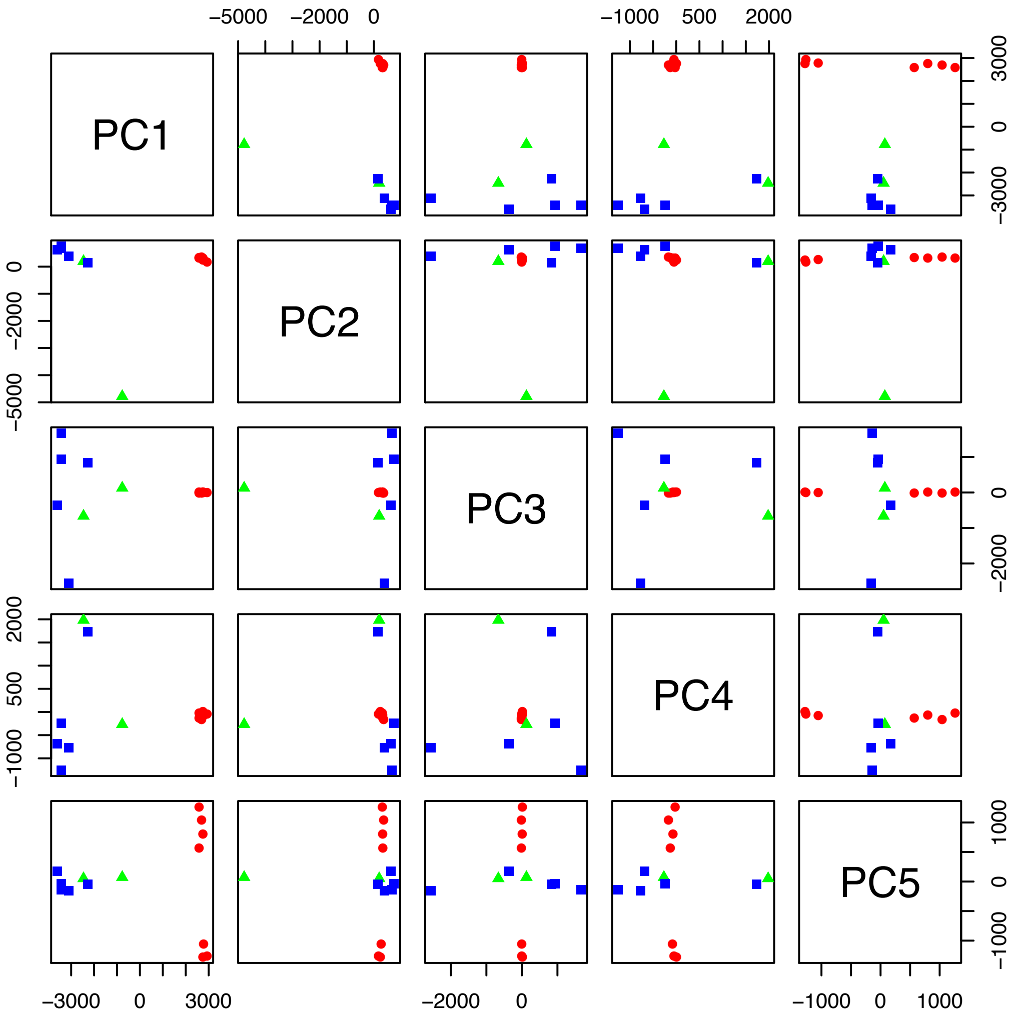

Suplemememtary Figure S4 Principal component analysis for the 13 *Capsicum* lines and ‘Takanotsume’.

C. annuum, *C. baccatum*, and *C. chinense* dots are shown in red, green, and blue, respectively.
